# The Menstrual Practices Questionnaire (MPQ): development, elaboration, and implications for future research

**DOI:** 10.1080/16549716.2020.1829402

**Published:** 2020-10-14

**Authors:** Julie Hennegan, Agnes Nansubuga, Agnes Akullo, Calum Smith, Kellogg J. Schwab

**Affiliations:** a The Water Institute, Department of Environmental Health and Engineering, Johns Hopkins Bloomberg School of Public Health, Baltimore, MD, USA; b Irise Institute East Africa, Jinja, Uganda; c Irise International, Sheffield, UK

**Keywords:** Menstrual health, outcome assessment, menstrual hygiene, reproductive health, women’s health, survey

## Abstract

High-quality evidence is needed to inform policies and programmes aiming to improve menstrual health. Quantitative studies must address the many evidence gaps in this field, and practitioners have increased monitoring and evaluation efforts to track their progress. A significant barrier to improving the rigor of this work is the lack of comprehensive and comparable measures to capture core concepts. The Menstrual Practices Questionnaire (MPQ) is a new tool to support comprehensive and standardised assessment of the activities undertaken in order to collect, contain, and remove menstrual blood from the body in self-report surveys. The questionnaire is freely available online for download and can be adapted for use across contexts and age groups. In this article, we describe the purpose of the MPQ as a best-practice tool to align the description of menstrual practices and provide a foundation for further question refinement. We outline the development of the tool using systematic review of qualitative studies of menstrual experiences, audit of measures used in the study of menstrual health and hygiene, survey of experts, insights from past research, and examples from piloted questions in a survey of adolescent girls in Soroti, Uganda. We describe the identification of menstrual practices as a priority for measurement, coverage of practices included in the MPQ, and justify the inclusion of location-specific questions. For each section of the questionnaire, we outline key reasons for the inclusion of practice items alongside elaboration for users to help inform item selection. Finally, we outline priorities for future research to refine the assessment and reporting of menstrual practices, including the identification of minimum reporting requirements for population characteristics to facilitate comparison across studies, testing the extent to which experiences during the most recent menstrual period reflect those over longer time periods, and further exploration of biases in self-report.

## Background

Increased acknowledgement that unmet menstrual health needs result in consequences for physical, mental, and social well-being has motivated policy and programme responses around the world [1–4]. However, there is a dearth of evidence to support these efforts [5,6]. Research is needed to understand menstrual experiences and inform the development of interventions, and to test and monitor their impacts. Quantitative methodologies are required to address many research questions, but have been limited by a lack of tools to measure core concepts [6,7].

To address this need we developed the Menstrual Practices Questionnaire (MPQ), which offers a comprehensive set of self-report questions to capture menstrual practices: all of the activities undertaken in order to collect, contain, and remove menstrual blood from the body. The MPQ draws on past research to provide a best-practice tool which can be refined through future work. This methods forum article presents: (1) rationale for the consistent assessment of menstrual practices; (2) the development of the MPQ including the coverage of questions and question formats (including recall period, location specificity, and use of single-, multiple-response or frequency questions) based on past research, expert input, and pilot survey in Uganda; (3) elaboration on each section of the MPQ to assist users to select and incorporate items in their work; and (4) directions for future research to improve the measurement and reporting of practice-related questions.

### Measuring menstrual practices

Menstrual practices shape the daily experience of menstruation [1]. As a result, they are frequently a topic for measurement. A recent systematic review auditing the measures used in trials of menstrual health interventions and their nested studies found menstrual practices were the most commonly measured concept, but that measurement was inconsistent with few studies measuring the same practices or using comparable questions [7].

Accurately and consistently measuring menstrual practices is crucial for many reasons:

First, to adequately assess population needs. Menstrual practices, such as the type of material used as absorbent, provide a picture of the population of study, context, and a reference point for understanding other needs. A lack of tools to guide practice measurement means practices can be unintentionally overlooked, resulting in an incomplete picture. Practice information combined with insights about population preferences can inform intervention approaches. For example, in a group expressing discomfort using cloth as menstrual absorbent, interventions providing alternative products may be indicated [5,8,9].

Second, consistently measuring and reporting menstrual practices is essential for considering external validity. That is, whether study results are likely to generalise to other contexts. For example, the effects of an intervention in which commercial menstrual pads are provided in a setting where most of the population uses cloth are unlikely to replicate in populations where such products are already widely accessible and used. Similarly, findings from observational studies describing the association between menstrual experiences and health or social outcomes may not generalise to populations with different practice profiles. Again, inconsistent or incomplete measurement risks the ability to compare and limits those using research evidence from appraising the relevance of a study for informing their work.

Third, studies may investigate the association between different menstrual practices and health, social or environmental outcomes. For example, studies may test the relationships between practices such as the frequency of changing menstrual materials and the risk of reproductive tract infections (RTIs) to inform self-care recommendations [10], or may estimate the impacts of disposal practices on sanitation systems [11].

Finally, where interventions seek to modify menstrual practices, research and monitoring will need to collect practice data to understand the implementation and success of these efforts.

Comprehensive and consistent measurement of menstrual practices is needed. The generation of new questions for each research or monitoring effort is likely to waste resources, and result in inconsistent and incomplete assessments. These motivations informed the development of the MPQ and should spur ongoing efforts to improve the quality and consistency of questions used.

## Development of the MPQ

### Methodological overview

Table 1 provides a summary of the research activities contributing to MPQ development.

**Table 1.** Overview of research activities contributing to the development of the MPQ.

Research activity and citation	Summary of participants and method
Systematic review and synthesis of qualitative studies of menstrual experiences [1]	Systematic searching identified 76 qualitative studies of women’s and girl’s menstrual experiences in low- and middle-income countries. The review synthesised findings across this body of evidence. In addition to the main synthesis, we recorded the menstrual practices reported across included studies.
Systematic review and audit of measures used in the study of menstrual health [7]	This effort audited the measures used in (1) trials of menstrual health interventions and studies nested within trials in LMICs, and (2) measure development studies which tested the reliability or validity of tools to measure menstrual experience from any country. Systematic searches identified 23 trials, 9 nested studies and 22 measure development studies.
Expert survey and consultation meeting	23 experts (52% researcher, 12% practitioner, 36% both) participated in an online survey in September 2018. Experts were invited for participation through emails to the *East and Southern Africa Menstrual Hygiene Management Research Network* and *MHM in 10 group* [12]. They provided feedback on priority practices for measurement, recall periods, and location dependency. Results of the survey were used as a foundation for discussion during a meeting of the *East and Southern Africa Menstrual Hygiene Management Research Network* meeting in October 2018 for further clarification and input.
Cross-sectional survey of schoolgirls in Soroti, Uganda [13]	A cross-sectional survey of 538 menstruating schoolgirls across 12 government schools in Soroti, Uganda was undertaken from March to May 2019. The mean age of participants was 14.49 (SD = 1.20) and 83% had gone without food, water, medicine or school supplies in the past year. Participants were selected systematically from Primary Levels 5 and 6, with additional recruitment in Levels 4 and 7 to achieve the final sample size which was based on the number of items tested for inclusion in the Menstrual Practice Needs Scale [13]. MPQ questions were included alongside the piloted scale items. Girls completed paper copies of the survey in English. Trained female enumerators provided verbal instructions and each question in Ateso (and English when helpful) in groups of no more than 6 girls.

### Availability

The Menstrual Practices Questionnaire (MPQ) is available for download from the Menstrual Practice Measures website (www.menstrualpracticemeasures.org). The tool is available under a Creative Commons Attribution-NonCommercial 4.0 International License and is free to download and use.

### Comprehensive assessment of menstrual practices

For the MPQ, we define menstrual practices as: all of the activities undertaken in order to collect, contain, and remove menstrual blood from the body. For some activities, the locations used are central to the task and are necessary to capture practices, such as the location for changing menstrual materials. Activities to collect and remove blood from the body necessarily include the cleaning and storage of menstrual materials.

**Figure 1.**
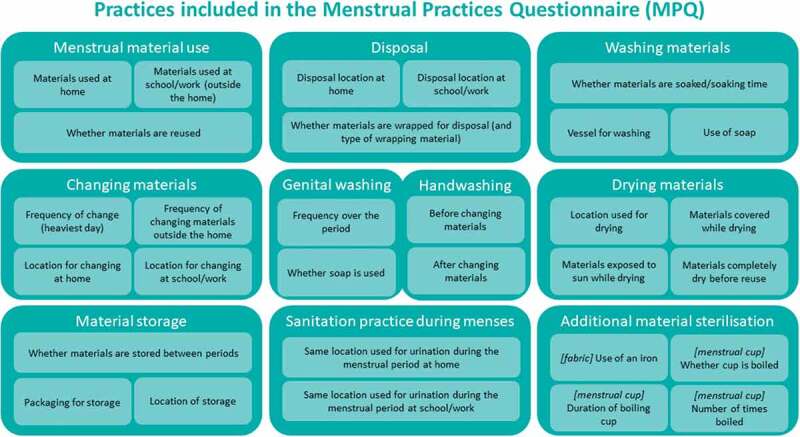
Summary of menstrual practices captured by items in the menstrual practices questionnaire (MPQ).

Figure 1 displays the practices included in the MPQ. Practices included in the measure were identified through a systematic review of qualitative studies of menstrual experiences. The review of studies from 35 low- and middle-income countries found menstrual practices were a key theme and extracted the types of practices described across studies. Three practices identified in the review were not included in the final tool. These were: whether materials were transported outside the home, the method of transporting materials (e.g. in a bag), and the water-source used for washing. Less than 50% of experts surveyed endorsed the usefulness of transport questions. The water-source for washing was excluded based on expert survey and consultation, with experts suggesting that soap use was more relevant for most studies. Practices identified through the qualitative review were cross-checked against those measured in past menstrual health trials, nested studies and measure development studies, extracted through a systematic review and audit of measures used in these studies [7]. All practices included in more than a single study were already included in the MPQ based on the qualitative review, except for the total number of materials used in a day. This question was excluded following expert survey in favour of items capturing the frequency of change. Questions about sterilisation practices, including ironing fabric materials and boiling menstrual cups were added based on the audit of past measures. Finally, questions capturing use of the usual urination location during menstruation were added based on recent studies emphasising the importance of this practice and the lack of available data [13,14].

### Recall period

**Figure 2.**
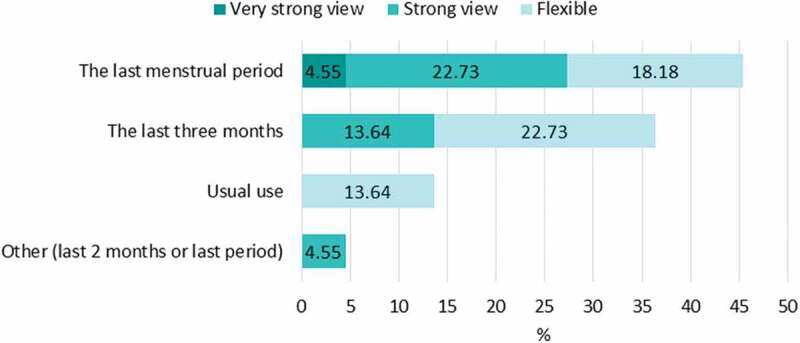
Surveyed experts’ views on the most appropriate recall period for self-reported menstrual practices (*n* = 22).

Questions in the MPQ relate specifically to the last menstrual period. This is the shortest possible recall period, and is designed to minimise recall bias [15]. In our survey of experts, this was the preferred recall period (see Figure 2).

Menstrual practices may vary over time based on evolving individual preferences, requirements of daily activities, and in response to changes in available resources and facilities, or menstrual cycle changes such as changes to menstrual flow due to contraceptive use. Particularly in monitoring or evaluating interventions, researchers will be interested in recent experiences to reflect uptake and changes in response to interventions. The last menstrual period was also selected to be comparable to national data. Nationally representative survey programs, including Performance Monitoring and Accountability 2020 (PMA2020), and the Multiple Cluster Indicator Surveys (MICS) have both collected data on menstrual experiences related to the last menstrual period [16,17].

### Location specificity

Menstruation occurs throughout the day and night making practices relevant across a range of locations. For those attending school or working outside of the home, many hours of the day are spent in these environments. Women and girls may use different menstrual practices outside the home to meet the demands of the work or school activities, due to social expectations, or available resources and facilities. Measuring location-specific menstrual practices provides specificity, ensuring the practices captured are most relevant to the outcome assessed, and identifying needs in different settings. For example, many menstrual health interventions focus on school environments. School-based practices are likely to be more closely related to school-based outcomes such as attendance or confidence than practices undertaken at home [5]. Data from our pilot study in Uganda are shown in Table 2, illustrating differences in practices between home and school environments.

**Table 2.** Proportion of respondents reporting using different menstrual practices according to location in a survey of schoolgirls in Soroti, Uganda (*n* = 538).

Menstrual practice	At home % (*n*)	At school % (*n*)
**Place used most often to change materials**		(*n* = 472)^a^
Latrine	19.89 (107)	51.83 (241)
Bedroom	52.42 (282)	–
Bathroom	26.39 (142)	35.05 (163)
Outside/in a garden	1.30 (7)	3.44 (16)
Another room (open text responses identified this as: teacher’s room, sick bay, unoccupied classroom)	–	9.68 (45)
*Missing*	*(0)*	*(7)*
**Type of material (grouped)**		
Cloth only	6.52 (35)	7.32 (39)
Disposable pad only (or tampon, *n* = 4)	37.24 (200)	43.53 (232)
Reusable pad only	16.95 (91)	16.32 (87)
Disposable and reusable pads	6.15 (33)	5.07 (27)
Disposable or reusable pads in combination with other materials (including cloth, toilet paper, cotton wool, mattress, underwear alone, natural materials)	23.09 (124)	13.51 (72)
Other materials only (toilet paper, cotton wool, mattress, underwear alone, natural materials)	10.06 (54)	14.26 (76)
*Missing*	*(1)*	*(5)*

^a^among those who changed materials outside the home

Not all questions in the MPQ are asked for both home and out-of-home environments. To balance comprehensiveness with participant fatigue and feasibility, practices most likely to differ across locations are asked separately.

### Single-response, multiple-response, and frequency questions

Many menstruators use multiple methods for each menstrual practice. For example, they use a variety of menstrual materials over their period. In collecting data to most accurately reflect menstrual practices, surveys may use multiple-response questions which record multiple behaviours, single response questions which force selection of only one response, or frequency-based questions which capture how often or in what proportion of instances respondents enacted a practice. There are strengths and weaknesses to each approach. The distribution of single-response, multi-response, and frequency-based questions in the MPQ represents our recommendations for best practice and offers a starting point to further investigate the performance of each approach in future studies. Where single-response questions are used, MPQ questions specify the practice used ‘most often’, acknowledging that participants may engage in more than one practice and supporting accurate data collection. More information related to each question is presented in the Question elaboration and guidance section.

### Question pilot and acceptability

MPQ questions were piloted as part of a cross-sectional survey of schoolgirls in Soroti, Uganda. In-depth training and translation with a group of six enumerators local to the area helped to refine question wording, and to test the comprehensibility of the questions. Training activities, translation from English to Ateso, and back translation activities including reframing questions in enumerators’ own words supported the interpretability of MPQ items, and refined questions for this context. Questions were well understood and inoffensive to the study population in Soroti, consistent with the high acceptability of menstrual-practice-focused questions in national surveys [16]. Users should consider context and language in adapting the questions to their needs. Many MPQ questions were piloted as multiple-response items in Soroti. Questions where low proportions of multiple responses were received in this group contributed to the final selection of multiple and single response items.

## Question elaboration and guidance

Practices captured in the MPQ are displayed in Figure 1 and specific questions presented in the MPQ (https://www.menstrualpracticemeasures.org/mpq/mpq-view-download/).

### Menstrual materials used

The type of menstrual material used was the most frequently assessed practice in past studies [7]. We recommend reporting material use as part of minimal description of population characteristics in menstrual health studies to aid comparability and appraisal of external validity. The MPQ captures the use of materials at home and away from home (school/work) separately, and we recommend this disaggregation where appropriate. National monitoring surveys currently measure all of the materials used without specifying location [16,18], MPQ data is comparable by combining at home and away from home responses.

The MPQ provides a question to differentiate the use of new or old cloth. This defines new cloth as that which was purchased to be used as menstrual absorbent, and old cloth as cloth used for something else first (e.g. clothing, bedding). This question may not be relevant for every program. In our survey of experts, 27% reported that it was ‘very important’ and 41% ‘important’ to distinguish between new and old cloth, while 32% rated this as ‘unimportant’. Our consultation meeting highlighted mixed perspectives; experts discussed that the disambiguation of new and old cloth may be useful to capture resource availability and financial stress, but that the way that cloth is cleaned is likely to be more relevant for those interested in hygiene or RTIs. The type of cloth may also be important for assessing environmental impacts.

The MPQ includes a dichotomous question to identify respondents who washed and reused materials rather than inferring reuse based on material type as diverse practices have been reported for materials such as cloth, foam, and cotton wool.

### Changing materials

The frequency of changing menstrual materials is likely to be dictated by the volume of bleeding, which varies across individuals and over the menstrual period. Failure to specify a time-period in past studies has made existing data difficult to interpret. The MPQ specifies the heaviest day of bleeding, ‘a day’ as 24 hours, and provides structured response options. This question may be useful alongside information about respondents’ preferences for changing, and reports of soiling. It may also be useful in studies investigating risk factors for genital irritation, such studies may need to incorporate additional questions about the frequency of change on other menstrual days where this may be more infrequent.

The location used to change menstrual materials at home provides an indication of the environments available, and a basis for assessing the acceptability of these environments for menstruators. PMA2020 has included this question in national monitoring surveys [19].

The frequency with which materials are changed outside the home is captured by the MPQ and can indicate the extent to which participants are willing to change outside the home and are impacted by the quality of school or workplace changing facilities. Identifying menstrual material change locations at school or work can be used in needs assessment in combination with information about the type of facilities available, and quantitative or qualitative data capturing participant perceptions of facilities [13,19]. These questions can also be used as part of assessing uptake and use of facilities where interventions seek to improve these services.

### Washing hands and genitals

In qualitative studies, women and girls have described the need to clean blood from hands and genitals to feel confident and comfortable during menses [1]. The MPQ offers questions to capture handwashing before and after changing materials which may be important for hygiene, particularly for populations using inserted materials such as tampons or menstrual cups [20]. Researchers should consider the relevance of these practices for their research questions. If observational data on handwashing facilities is available, this may be more relevant for describing populations for cross-study comparison. The questionnaire does not include whether soap was used for handwashing, and this addition should be considered where appropriate to the research question.

Population preferences and beliefs regarding genital washing vary widely [1]. MPQ questions focus on genital washing, rather than bathing the whole body as qualitative research has emphasised challenges related to washing away menstrual blood from the genitals and studies of menstrual practice-related irritation are most likely to be concerned with genital washing [10]. In evaluating interventions that may provide female-friendly facilities where regular genital washing is preferred, these questions may be used to assess implementation and uptake [21].

### Disposal

Menstrual waste disposal has been reported to vary in response to privacy concerns, taboos, and available facilities and services. We recommend describing disposal practices whenever reporting on population characteristics in menstrual health research. Both PMA2020 and MICS national data capture disposal practices, but do not specify a location. It is likely this captures practices at home, the location respondents spend much of their time and place the survey is administered [16,22]. The MPQ separately captures disposal at home and away from home (at school or work) to provide insights on these settings. This is likely to be particularly relevant where interventions target certain environments. Menstrual waste can quickly fill or damage sanitation systems. Studies evaluating the environmental or waste management implications of menstrual practices should include the MPQ question assessing whether materials were wrapped in anything else for disposal. Pilot data collection in Soroti, Uganda found that 74% of girls wrapped their materials for disposal, with 45% wrapping in plastic.

### Storage

Whether materials are stored between periods provides insights about the availability of storage spaces, the duration of reuse of reusable materials, and availability of materials. Having acceptable storage spaces is important for considering the feasibility of reusable material provision. The location of storage has also been implicated in *Candida* infection in one cross-sectional study [10]. No relationship was found between the type of packaging used to store materials in this study; however, packaging may be explored in additional studies, provide useful context for understanding the location of storage, and is included in the MPQ for use as needed. Open text questions included in pilot data collection in Soroti, Uganda, and past research [10], helped to identify response options for this question.

### Washing materials

Soaking is included in the MPQ to indicate the amount of time that a washing basin or bucket may be in use and to contextualise concerns around privacy while washing materials. In pilot data collection in Soroti, Uganda, 61% of those reusing materials reported soaking these for 10 minutes or longer, with 26% soaking for between one and two hours. This significantly changes our understanding of reported privacy concerns around washing. The use of a shared or personal vessel for washing similarly provides an understanding of women’s and girls’ experience and the constraints on their washing behaviour. Finally, the use of soap for washing is frequently included in studies testing associations between hygiene practices and RTIs [10,23], and can help indicate the safety of reused materials or adherence to hygiene guidance where this is provided in interventions.

### Drying materials

Questions included in the MPQ offer a comprehensive assessment of drying practices, specifying a range of locations for drying. These are drawn from experiences across multiple past surveys [24,25] categorising locations as outside or inside, and as hanging (openly) or hidden. In addition to the location for drying, the MPQ provides questions to capture covering materials, exposure to sunlight and reuse of materials that have not fully dried. Exposure to sunlight and the use of completely dry materials have been emphasised for hygiene [10,25]. However, asking only if materials are dried in the sun may fail to capture exposure to UV light if materials are covered to protect privacy. In our pilot survey in Soroti, 55% of schoolgirls covered materials while they were drying, including 57% of those who reported drying in the sunlight ‘every time’ and 58% of those who reported drying in the sunlight ‘sometimes’.

### Sterilising menstrual materials

Washing and drying are not the only relevant practices to cleaning menstrual materials. Some fabric materials can be ironed, while many menstrual cups should be boiled for sterilisation. Ironing fabric should be considered in combination with washing and drying practices when assessing hygiene practices and risks of RTIs. Further, some commercial reusable pads include instructions that they should not be ironed. In our pilot survey in Soroti, Uganda, we found that a total 33% of those reusing materials ironed these (including 35% of those using reusable pads, and 24% of cloth users). Current ironing practices can inform guidance for reusable product interventions and adherence to guidance. Boiling practices will provide insights into uptake and adherences of menstrual cup cleaning practices [26].

### Toilet/latrine use during menstruation

Although urination behaviours during menstruation do not strictly represent blood containment or removal, the presence of menses has been overlooked in contributing to sanitation behaviours. Studies in India, and our pilot survey in Uganda, suggest that menstruation may alter sanitation behaviours and that these should be considered in capturing practices during menstruation. The Menstrual Practice Needs Scale, developed at the same time as the MPQ found that in Soroti, Uganda, only 27% of schoolgirls surveyed indicated they were ‘always’ comfortable urinating in their usual location at home during their last menstruation, while 37% were never comfortable [13]. In expanding the conceptualisation of menstrual hygiene to more comprehensively capture needs, MacRae et al. [14] included supportive spaces for urination and defecation while menstruating. We recommend studies assessing menstrual needs to consider urination behaviour during menstruation.

## Implications for future research

The quality of self-reported measures for capturing menstrual practices and other core concepts in menstrual health research will be critical to high-quality quantitative research in this field. The sensitive nature of menstrual behaviours precludes observational data collection for many practices and there has been limited funding to evaluate the performance of current questions. The MPQ offers a first step for improving comparability.

The MPQ offers a comprehensive selection of practice questions. However, future consensus efforts are needed to identify minimum reporting requirements for describing populations in menstrual health studies. As highlighted in the introduction, it will be difficult to appraise studies’ external validity and synthesise insights across the evidence base if key practice information is missing. As practices of interest will differ according to research objectives, a shorter set of core items for population description will facilitate comparability.

More research is needed to understand and minimise bias in self-reported menstrual practices. For many practices of interest, self-reported will be the only feasible means of data collection. While observational methods may be used to identify the available facilities and services such as sanitation, washing or disposal facilities, or the cost and accessibility of menstrual materials from local vendors, this information will be unable to tell us what women in the population of interest do. Self-reported data will frequently be required but is subject to a myriad of biases. Assessments of test-retest reliability and comparisons between retrospective report and diary records are needed to understand the reliability of questions and improve their performance.

The MPQ uses the last menstrual period as the recall time frame. This was selected to minimise recall bias and participant burden involved in recalling multiple periods. However, research is needed to understand the extent to which the most recent period reflects menstrual practices over longer time periods (for example, practice over the past 6 months). Future research should explore this topic to provide guidance for study and national-level measurement. For example, condom use at last sex has been found to be an acceptable proxy for use over longer recall periods such as the last 14 or 60 days [27]. Assessment of practices at the last menstrual period may introduce variability due to one-off experiences and may be more susceptible to seasonal variations or fluctuations in attendance at school or work for these questions (for example, recent school holidays). At the same time, longer time periods risk respondents minimising variability across experiences and providing more socially desirable responses as their ‘average’ practice.

More research is needed to provide guidance on the association between menstrual practices and risks of RTIs [28]. This may also indicate modifications to the MPQ and practice assessment where practices associated with fewer risks are identified. For example, future research may identify minimal changing recommendations for menstrual materials, or exposure to UV or other sterilisation practices which may be used to refine the measurement and reporting.

It is important to note that in providing the MPQ we do not suggest that menstrual practices are the most important topic of study for menstrual health. Practices should be measured and reported for population description across studies, but other constructs may provide greater insights into menstrual experience. Menstrual practices have been the most reported concept in menstrual health trials and nested studies [7]. This speaks to their value in participant description and research questions which have focused on describing population practices and variability across settings such as urban and rural environments [29]. However, qualitative meta-synthesis of extant studies of menstrual experience found menstrual practices to be only one theme among many reflecting menstrual experience and antecedents contributing to this experience. Understanding the extent to which women and girls perceive their own needs as being met, as captured by the recent Menstrual Practice Needs Scale [13], or consequences for stress and school participation related to menstruation [30] may represent more important concepts for many research questions and for national monitoring where useful indicators are identified. MPQ items can be used in combination with such measures to provide a detailed understanding of experiences and their association with physical, mental, and social well-being.

## Conclusions

The MPQ offers a comprehensive set of questions for the measurement of menstrual practices. It is a flexible tool and can be used as a foundation for researchers developing their own surveys to select practices most relevant to their research questions. In providing a thorough base, the MPQ encourages users to consider a wide range of practices, including those which have been overlooked to the detriment of the evidence base. Further, it reduces the burden of generating comprehensive survey items comparable to past research. MPQ items have been piloted in data collection with adolescent girls, and questions can be modified for contextual needs and use in adult populations. The tool will support the comparability of menstrual practice data collected over time and provide opportunities to further interrogate the performance of practice-related questions so they can be refined.

## Paper context

A major barrier to quantitative research to inform practice and improve menstrual health has been the lack of quality measurement tools. This paper shares the Menstrual Practices Questionnaire which offers a comprehensive set of self-report questions to capture menstrual management behaviours. Use of the tool will improve the quality of needs assessments, support studies testing relationships between menstrual behaviours and health outcomes, and ensure external validity can be meaningfully considered across menstrual health studies.
